# CD8^+^ T Cell Response Quality Is Related to Parasite Control in an Animal Model of Single and Mixed Chronic *Trypanosoma cruzi* Infections

**DOI:** 10.3389/fcimb.2021.723121

**Published:** 2021-10-12

**Authors:** Jose Mateus, Paola Nocua, Paola Lasso, Manuel Carlos López, M. Carmen Thomas, Adriana Egui, Claudia Cuervo, John Mario González, Concepción J. Puerta, Adriana Cuéllar

**Affiliations:** ^1^ Grupo de Enfermedades Infecciosas, Facultad de Ciencias, Pontificia Universidad Javeriana, Bogotá, Colombia; ^2^ Grupo de Inmunobiología y Biología Celular, Facultad de Ciencias, Pontificia Universidad Javeriana, Bogotá, Colombia; ^3^ Instituto de Parasitología y Biomedicina López Neyra, Consejo Superior de Investigaciones Científicas, Granada, Spain; ^4^ Grupo de Ciencias Básicas Médicas, Facultad de Medicina, Universidad de los Andes, Bogotá, Colombia; ^5^ Grupo de Ciencias de Laboratorio Clínico, Facultad de Ciencias, Pontificia Universidad Javeriana, Bogotá, Colombia

**Keywords:** T cells, *Trypanosoma cruzi*, Chagas disease, reinfections, immune quality

## Abstract

Chagas disease (ChD) is a chronic infection caused by *Trypanosoma cruzi*. This highly diverse intracellular parasite is classified into seven genotypes or discrete typing units (DTUs) and they overlap in geographic ranges, vectors, and clinical characteristics. Although studies have suggested that ChD progression is due to a decline in the immune response quality, a direct relationship between T cell responses and disease outcome is still unclear. To investigate the relationship between parasite control and immune T cell responses, we used two distinct infection approaches in an animal model to explore the histological and parasitological outcomes and dissect the T cell responses in *T. cruzi*-infected mice. First, we performed single infection experiments with DA (TcI) or Y (TcII) *T. cruzi* strains to compare the infection outcomes and evaluate its relationship with the T cell response. Second, because infections with diverse *T. cruzi* genotypes can occur in naturally infected individuals, mice were infected with the Y or DA strain and subsequently reinfected with the Y strain. We found different infection outcomes in the two infection approaches used. The single chronic infection showed differences in the inflammatory infiltrate level, while mixed chronic infection by different *T. cruzi* DTUs showed dissimilarities in the parasite loads. Chronically infected mice with a low inflammatory infiltrate (DA-infected mice) or low parasitemia and parasitism (Y/Y-infected mice) showed increases in early-differentiated CD8^+^ T cells, a multifunctional T cell response and lower expression of inhibitory receptors on CD8^+^ T cells. In contrast, infected mice with a high inflammatory infiltrate (Y-infected mice) or high parasitemia and parasitism (DA/Y-infected mice) showed a CD8^+^ T cell response distinguished by an increase in late-differentiated cells, a monofunctional response, and enhanced expression of inhibitory receptors. Overall, our results demonstrated that the infection outcomes caused by single or mixed *T. cruzi* infection with different genotypes induce a differential immune CD8^+^ T cell response quality. These findings suggest that the CD8^+^ T cell response might dictate differences in the infection outcomes at the chronic *T. cruzi* stage. This study shows that the T cell response quality is related to parasite control during chronic *T. cruzi* infection.

## Introduction

The T cell responses induced after infection or vaccination might be used as a possible predictor of protection against pathogens. Although T cells are heterogeneous, their immune quality is defined as the efficient maintenance of memory T phenotypes and a competent antigen-specific T cell response (Appay et al., 2002; Darrah et al., 2007; Seder and Darrah, 2008; Riou et al., 2012; Ferreira et al., 2019). Efficient maintenance of early memory T cell subsets has been associated with the protection induced by successful vaccination and infection models (Ribeiro et al., 2014; Vigano et al., 2015; Akondy et al., 2017; Mpande et al., 2018). Memory T cell subsets encompass populations with distinct levels of differentiation, including central memory (T_CM_), effector memory (T_EM_), and effector cells (Brummelman et al., 2018). Early differentiated stem memory cells (_TSCM_), an antigen-experienced T cell subset endowed with the ability to self-renew and reconstitute memory and effector T phenotypes, were recently described (Gattinoni et al., 2009; Gattinoni et al., 2011; Brummelman et al., 2018). Additionally, a competent cellular response is defined as the capacity of individual T cells to simultaneously produce several cytokines or molecules participating in the cytotoxic response (Darrah et al., 2007; Westerhof et al., 2019), and this feature is affected by the expression of inhibitory molecules, such as programmed cell death-1 (PD-1 or CD279) and cytotoxic T-lymphocyte–associated antigen 4 (CTLA-4 or CD152) (Wherry, 2011; Wherry and Kurachi, 2015). Studies in diseases caused by viruses, bacteria, fungi, or protozoa have shown that the lack of infection control is associated with an increase in the percentages of late-differentiated T cells, the loss of functional T cell capacities, and an increase in immune inhibition due to augmented expression of inhibitory molecules on T cells (Darrah et al., 2007; Gigley et al., 2012; Attanasio and Wherry, 2016; McLane et al., 2019). Indeed, similar findings from studies on T cells in cancer have led to the development of immunotherapy with antibodies against inhibitory molecules such as anti-PD-1 or anti-CTLA-4. A treatment that reverses the observed impairments in the immune response quality and boosts the antigen-specific T cell response (Moreira et al., 2020).

Protozoan infections are responsible for many fatal human diseases in undeveloped countries, including malaria, sleeping sickness, visceral leishmaniasis, and Chagas disease (ChD) (Barrett et al., 2019). *Trypanosoma cruzi*, the causal agent of ChD, is an intracellular microorganism with a predilection for cardiac muscle or gastrointestinal tract tissues that can cause serious human pathologies such as cardiomyopathy or megasyndromes in chronically infected patients (Perez-Molina and Molina, 2018; WHO, 2020). *T. cruzi* exhibits high genetic diversity, which has led to classification into seven genotypes or discrete typing units (DTUs, TcI-TcVI and TcBat) based on genetic markers. The parasite genotypes show overlaps in their geographic ranges, vectors, and clinical characteristics (Messenger et al., 2015; Zingales, 2018). A comprehensive study that included several genetic *T. cruzi* groups found strains from different genotypes induced a high degree of heterogeneity in inflammation degree or immune response in acutely and chronically infected mice (Santi-Rocca et al., 2017). For example, TcI and TcII strains, which are the most predominant *T. cruzi* genotypes in Latin America, have shown high diversity in inflammation outcomes ranging from mild to severe both chagasic patients and animal models (Barrera et al., 2008; Cruz et al., 2015; Sales-Campos et al., 2015; Hernandez et al., 2016; Zingales, 2018; Ledezma et al., 2020). Indeed, epidemiological studies of individuals with positive Chagas serological tests have shown that continuous exposure to infections with distinct *T. cruzi* genotypes increases the risk of progression to chronic Chagas cardiomyopathy (Zicker et al., 1990; Basquiera et al., 2003; Sabino et al., 2013; Perez et al., 2014). Similar results have been observed in animal models of ChD, which revealed that reinfections with *T. cruzi* strains might determine the severity of cardiac damage (Bustamante et al., 2002; Bustamante et al., 2007). However, although various studies have investigated chronic ChD, it is unknown why approximately 30-40% of *T. cruzi*-infected patients develop cardiac or gastrointestinal illnesses decades after the initial infection (Acevedo et al., 2018; Bonney et al., 2019).

A strong effector T cell response is induced by *T. cruzi* infection, and this response results in the secretion of cytokines and the release of cytotoxic granules upon antigen recognition. Several lines of evidence indicate that T lymphocyte type I responses (T helper (Th) or T cytotoxic (Tc) type I responses) are the central mediator of protection against *T. cruzi* infection; however, Th17 and regulatory T cell (Treg) responses might influence the infection outcome (Cai et al., 2016; Ersching et al., 2016; Araujo Furlan et al., 2018). Additionally, studies in humans and mice have shown that chronic *T. cruzi* infection leads to a decay in the T cell response quality accompanied by a marked increase in late-differentiated T cells, a decreased multifunctional T cell capacity, and higher expression of inhibitory receptors (Laucella et al., 2004; Albareda et al., 2013; Lasso et al., 2015; Mateus et al., 2015), suggesting that T cells might be associated with the progression of disease severity. Indeed, the administration of antiparasitic therapy to individuals with chronic *T. cruzi* infection and in mouse models of chronic *T. cruzi* infection has shown that treatment reverses the deterioration of the T cell response (Bustamante et al., 2008; Vallejo et al., 2016; Mateus et al., 2017; Perez-Anton et al., 2018; Egui et al., 2020). Overall, although the mechanisms involved in the pathogenesis of ChD are unknown, the available evidence suggests that parasite persistence drives the impairment of the T cell response and leads to uncontrolled *T. cruzi* infection and progression of the disease.

Several studies in Chagasic patients or animal models with chronic *T. cruzi* infection have suggested a relationship between T cell responses and ChD severity (Pinazo et al., 2015; Cortes-Serra et al., 2020). However, validating this hypothesis has been difficult because of the natural ChD history, which makes the long-term follow-up of patients for decades after the initial infection complex. Thus, to investigate whether the infection outcome is related to the T cell response quality during chronic *T. cruzi* infection, we used two distinct infection approaches in an animal model to explore the histological and parasitological outcomes and dissect the T cell responses in *T. cruzi*-infected mice. We examined the T cell response focused on the following parameters: memory/effector subsets, antigen-specific response, and expression of inhibitory receptors on T cells. First, we performed a set of single infection experiments with *T. cruzi* DA (TcI) or Y (TcII) strains to compare the infection outcomes and evaluate the relationship with the T cell responses in acute and chronic parasite infection. We selected these two *T. cruzi* strains since they represent the most predominant genetic groups in Latin-American countries and have shown different inflammatory profiles and infection outcomes in animal models (Santi-Rocca et al., 2017; Zingales, 2018). Next, because infections with diverse *T. cruzi* genotypes can feasibly occur in naturally infected individuals, mice were infected with either the Y or DA strains and subsequently reinfected with the Y strain, and the mice with homologous or heterologous infection with *T. cruzi* strains (Y/Y or DA/Y) were assessed to determine the relationship of the infection outcome with the T cell response.

## Materials and Methods

### Mice and Parasites

Female inbred BALB/cAnNCr mice (aged 6 to 8 weeks) were purchased from Charles River Laboratories International, Inc. (Wilmington, MA, USA) and housed in specific pathogen-free (SPF) animal facilities from the Unidad de Biología Comparativa at the Pontificia Universidad Javeriana. The BALB/c mouse strain was selected to minimize variability compared with previous studies (Hoft and Eickhoff, 2002; Mariano et al., 2008; Sanoja et al., 2013; Egui et al., 2017; Mateus et al., 2019). The animals were housed in polycarbonate cages (4 or 5 animals/cage) with sterile soft wood shaving bedding, which was changed weekly, and these cages were maintained in ventilated racks in an animal biosafety level 2 (ABSL-2) room under constant noise-free environmental conditions. The mice received filtered water (changed weekly) and a standard mouse maintenance diet *ad libitum*. Stress and microbiological monitoring (including behavioral and animal welfare analyses and microbiological and serological testing) were performed according to IACUC guidelines. *T. cruzi* trypomastigotes from the DA (MHOM/CO/01/DA; discrete typing unit (DTU) TcI) or Y strain (MHOM/BR/00/Y; TcII) were used in the present study. Trypomastigotes of both strains were maintained by tissue culturing involving serial passage through a monolayer of renal fibroblast-like cells or VERO cells (ATCC CCL-81, Manassas, VA, USA). Trypomastigotes of the Y strain were passaged in female inbred BALB/cAnNCr mice at least three times to maintain virulence. Both *T. cruzi* strains represent the most predominant genetic groups in Latin America and have shown different inflammatory profiles and infection outcomes in animal models (Barrera et al., 2008; Cruz et al., 2015; Sales-Campos et al., 2015; Santi-Rocca et al., 2017; Zingales, 2018; Ledezma et al., 2020). For instance, an infection with the *T. cruzi* DA strain has shown a prolonged infection with preferential migration to cardiac tissue and reappearance of parasitemia in chronic stages of infection (Barrera et al., 2008; Cruz et al., 2015). In contrast, strain Y shows an aggressive illness with high parasitemia levels in the acute phase and tropisms towards the liver and colon (Mateus et al., 2019).

### Infection and Challenges Experiments in Mice

Forty BALB/c mice were randomly divided into two experimental groups and infected intraperitoneally (i.p.) with 10^5^ trypomastigotes of the Y or DA strain in 100 μl of PBS under aseptic conditions. Five mice per group were euthanized by CO_2_ inhalation at 10, 30, 100, or 260 days postinfection (dpi). We selected the time periods for the acute (10 and 30 dpi) or chronic phases (100 and 260 dpi) based on a previous study carried out by our research group that demonstrated differential infection outcomes and T cell immune responses in *T. cruzi*-infected mice (Mateus et al., 2019). This is in the sense that we and others evaluate in similar days post-infection the acute and chronic infection stages evaluating immunological parameters of *T. cruzi*-infected mice (Bustamante et al., 2002; Hoft and Eickhoff, 2002; Bustamante et al., 2007; Bustamante et al., 2008; Sanoja et al., 2013; Mateus et al., 2019).

For the assessment of homologous and heterologous infection with *T. cruzi* strains, 20 BALB/c mice were randomly divided into two experimental groups and infected i.p. with trypomastigotes of either the DA or Y strain under the above-mentioned conditions, and five mice per group were then challenged at 10 or 100 dpi with 10^5^ trypomastigotes of the Y strain. Both mouse groups were euthanized by CO_2_ inhalation at 260 dpi as described above. We obtained spleen samples from all the mice for cell purification and samples of the cardiac blood, skeletal muscle of the posterior leg, heart, colon and liver tissue for DNA extraction or histopathology analyses. The sample size was determined based on the average number of mice used in previous studies of *T. cruzi* infection in mice (Maranon et al., 2001; Planelles et al., 2001; Egui et al., 2012; Sanoja et al., 2013). Various animal welfare indicators (score sheet) were recorded weekly during the first 30 days and then every month thereafter. One experiment was conducted to evaluate either single or mixed *T. cruzi* infections in mice. Each mouse group included five biological replicates. If the mouse groups were pooled, each group included nine or ten biological replicates. Each figure legend describes the number of mice included in each group.

### *T. cruzi* Soluble Antigens

The *T. cruzi* soluble antigens (*Tc*SA) were obtained from the Y strain using previously described methods (Martinez-Calvillo et al., 2007; Fernandez-Villegas et al., 2011; Mateus et al., 2013). Briefly, amastigotes and trypomastigotes (1:1 ratio) were collected from the VERO cell culture supernatants at 96-120 hours postinfection (Fernandez-Villegas et al., 2011). The parasites were then washed twice with cold 1× PBS (Eurobio) and resuspended at a density of 1 x 10^6^ parasites/μl in lysis buffer as previously reported (Martinez-Calvillo et al., 2007). The parasites were incubated on ice for 30 minutes, and supernatants containing *Tc*SA were collected by centrifugation at 12,000 x *g* and 4°C for 15 minutes and stored at -80°C until use. The protein concentrations were determined using the Bradford assay, and the protein profiles were analyzed by SDS-PAGE followed by Coomassie blue staining (Gibco BRL; Grand Island, NY, USA). Noninfected VERO cell supernatants were subjected to all above-described procedures and used as a mock control in the flow cytometry assays.

### Flow Cytometry

The antibodies used in the present study are listed in **Supplemental Table 1**. The Fixable Aqua Dead Cell Stain viability marker (LIVE/DEAD) (Invitrogen; Eugene, OR, USA) was used to exclude dead cells. All conjugated antibodies were titrated and evaluated using FMO controls as previously described (Mateus et al., 2013). Spleen cells were plated in 96-well round-bottom tissue culture plates and stained with multicolor immunofluorescence panels for assessment of CD4^+^ and CD8^+^ T cell responses.

First, cells were treated with Fc block antibodies (BD Biosciences) for 5 minutes at 4°C and subsequently stained with the LIVE/DEAD marker for 20 minutes at room temperature. To evaluate the memory/effector phenotypes, the cells were stained with antibodies against CD3, CD4, CD8, CD44, CD62L, CD122, CD127, and KLRG1 antigens for 30 minutes at 4°C. To evaluate antigen-specific T cell-producing cytokines, the cells were cultured with mock (as a negative control) and *Tc*SA (1 µg/ml) in the presence of anti-CD28 (1 μg/ml, clone 37.51, BD Pharmingen) for 1 hour at 37°C in a humidified atmosphere containing 5% CO_2_ and then incubated in the presence of brefeldin A (1 μg/ml) and monensin (0.7 μg/ml) (BD Biosciences) for 5 hours. After incubation, the cells were stained with the viability marker and then with antibodies against CD3, CD4, and CD8 molecules for 30 minutes at 4°C and washed with staining buffer. The cells were fixed and permeabilized with Cytofix/Cytoperm buffer (BD Biosciences) according to the manufacturer’s instructions and incubated with anti-IFNγ, anti-TNFα, and anti-IL-2 antibodies for 30 minutes at 4°C. To evaluate the functional subsets of CD4^+^ T cells, the cells were cultured under the above-described conditions, stained with viability markers and then with antibodies against CD3, CD4, CD25, and LAP markers for 30 minutes at 4°C and washed with staining buffer. The cells were fixed and permeabilized with Foxp3 Transcription Factor Fixation/Permeabilization (Invitrogen) according to the manufacturer’s instructions and incubated with anti-Foxp3, anti-RORγt, anti-IL-10, anti-IL17A, and anti-IL-21 antibodies for 30 minutes at 4°C. To evaluate the expression of inhibitory receptors on T cells, the cells were stained with antibodies against CD3, CD4, CD8, PD-1 (CD279), 2B4 (CD244), and CD160. The cells were then fixed and permeated using Cytofix/Cytoperm buffer according to the manufacturer’s instructions and incubated with anti-CTLA-4 (CD152) antibody for 30 minutes at 4°C.

At least 50,000 events gated on live CD3^+^ cells were acquired with a FACS Aria II flow cytometer (BD Biosciences). **Supplemental Table 2** provides the cell counts collected for the CD3^+^CD4^+^ and CD3^+^CD8^+^ cells. The data were analyzed using FlowJo 9.3 (Tree Star; Ashland, OR, USA), Pestle 1.7 (National Institutes of Health (NIH), Bethesda, MD, USA), and SPICE 5.3 (NIH) software. A Boolean analysis was performed to define the multifunctional profiles and the coexpression of inhibitory receptors on FlowJo. The Boolean analysis for the multifunctional profiles included IFNγ, TNFα, and IL-2 and for the coexpression of inhibitory receptors included PD-1, 2B4, CD160, and CTLA-4 gated on CD4^+^ and CD8^+^ T cells. Dead and doublet cells were excluded from the analysis (**Supplemental Figure 1**). A positive cytokine response was defined for each measured profile, and this response was determined as the median frequency of the T cell response obtained from uninfected mice after stimulation with *Tc*SA plus 1 SD after background subtraction (cells from each mouse cultured with Mock).

### Parasite Quantification by qPCR

Sample tissues from each mouse were collected and processed as described previously (Mateus et al., 2019). Briefly, DNA from tissue samples was extracted using a High Pure PCR template preparation kit according to the manufacturer’s instructions (Roche, Mannheim, Germany). For the assessment of DNA integrity and to exclude the presence of inhibitors in the sample, PCR was then performed using the CytB Uni fw 5’-TCATCMTGATGAAAYTTYGG-3’ and CytB Uni rev 5’-ACTGGYTGDCCBCCRATTCA-3’ primers, which amplify the cytochrome B gene of small mammalian species, as described previously (Schlegel et al., 2012). Subsequently, qPCR was performed with the Cruzi 1 5’-ASTCGGCTGATCGTTTTCGA-3’ and Cruzi 2 5’-AATTCCTCCAAGCAGCGGATA-3’ primers and the Cruzi 3 5’-6FAM-CACACACTGGACACCAA-BBQ-3’ probe, which amplify a 166-bp segment of *T. cruzi* satellite DNA (Piron et al., 2007). Each sample was analyzed in duplicate. The parasite load was estimated based on a standard curve and constructed with different DNA concentrations of the Y strain mixed with 50 ng of DNA from tissue sampled from an uninfected mouse, which ranged from 10^-1^ – 10^4^ parasite equivalents per 50 ng of DNA as described previously (Cummings and Tarleton, 2003; Mateus et al., 2019). Parasite loads below the limit of quantification (LOQ) were set to LOQ/2 (0.05 parasite equivalents per 50 ng of DNA) as previously described (Hecht et al., 2018). Amplification was performed using the Applied Biosystems™ QuantStudio™ 3 Real-Time PCR System (Applied Biosystems, USA) and previously described qPCR conditions (Duffy et al., 2013). Each PCR performed in this analysis included the following controls: reaction (water added in the room containing the reaction mixture), gray (water added in the room where the sample was added to the reaction), negative (genomic DNA from one uninfected mouse), and positive (DNA from the DA and Y strains).

### Histopathology

Sample tissues from each mouse were stained with hematoxylin and eosin (H&E) for blinded analysis according to the following features: presence of inflammation, type of cellular infiltration, and pathological changes. Histopathological scores were assigned as follows using previously described methods (Guarner et al., 2001): absent, mild, moderate, or severe. Representative pictures of different inflammatory infiltrate scores observed in tissue samples from DA and Y-infected mice are shown in **Supplemental Figure 2**.

### Statistical Analysis

Statistical analyses between two groups were performed using the Mann–Whitney U test. The correlations between the T cell response and the parasite loads in tissue were analyzed using Spearman’s rank correlation coefficient. The tests were two-tailed, and *p*<0.05 indicated statistical significance. GraphPad Prism 8.0 for Mac OS X software (GraphPad, San Diego, CA, USA) was used for the statistical analyses.

## Results

### Distinctive Outcomes in Mice With Acute and Chronic Infections With *T. cruzi* Y or DA Strains

**Figure 1:**
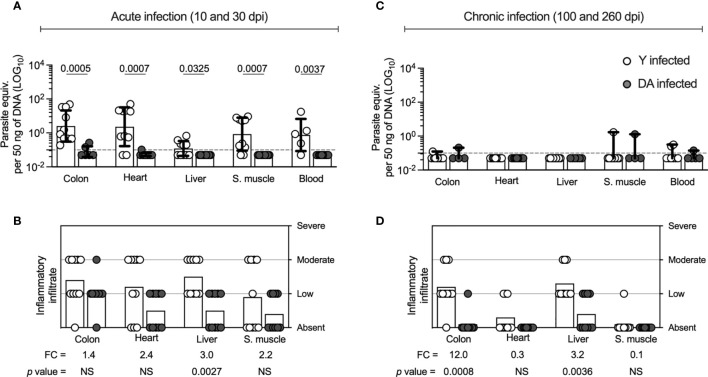
Parasite loads and inflammatory infiltrate scores of tissues from Y- or DA-infected mice. **(A, C)** Parasite loads in colon, heart, liver, skeletal muscle, and blood samples from acutely and chronically Y- or DA-infected mice, respectively. The bar graphs show the medians and ranges of the parasite equivalent per 50 ng of DNA (LOG_10_) in tissues from each group of infected mice. The dotted line represents the cutoff for the limit of detectable quantification (LOQ) based on serially diluted *T. cruzi*-spiked tissue DNA as described in the Materials and Methods (0.1 parasite equivalents per 50 ng of DNA). **(B, D)** Inflammatory infiltrate scores obtained for colon, heart, liver, and skeletal muscle samples from the acutely and chronically Y- or DA-infected mice, respectively. The bar graphs show the average inflammatory infiltrate scores found for the tissues from each group of infected mice. The fold change (FC) shown in B and C was determined as the average inflammatory score detected in the Y-infected mice divided by the average infiltrate inflammatory score found in the DA-infected mice. Each point represents the value of the parasite load or the infiltrate inflammatory score detected in each mouse infected with the Y (white) or DA (gray) strain of *T. cruzi*. Results are pooled from one experiment with ten mice per group. The *p* values were calculated using the Mann–Whitney U test.

Because several studies have documented that the genetics and phenotypic heterogeneity of *T. cruzi* strains may be associated with distinct infection outcomes (Santi-Rocca et al., 2017; Zingales, 2018), we first analyzed the parasitological and histological outcomes during *T. cruzi* infection in experimentally infected mice with *T. cruzi* trypomastigotes belonging to two strains of different genotypes (Y or DA) as described in the *Materials and Methods* section. The Y and DA strains were isolated from infected patients and belong to the TcII and TcI genotypes of *T. cruzi*, respectively (Pinto et al., 1999; Barrera et al., 2008; Amato Neto, 2010; Cruz et al., 2015). We pooled acutely and chronically infected mice from days 10 and 30 and days 100 and 260, respectively, as described previously (Mateus et al., 2019). The parasite load and the inflammatory infiltrate scores in tissue samples corresponded to the infection outcomes evaluated in the acutely and chronically DA- or Y-infected mice. The colon (*p* = 0.0005), heart (*p* = 0.0007), liver (*p* = 0.0325), skeletal muscle (*p* = 0.0007), and blood (*p* = 0.0037) samples from the acutely Y-infected mice showed significantly higher parasite loads than those from the acutely DA-infected mice (**Figure 1A**). We found a greater inflammatory infiltrate score in liver samples (*p* = 0.0027) from Y-infected mice than in DA-infected mice (**Figure 1B**). In chronically infected mice, the parasite loads in tissues were similar in samples from Y- or DA-infected mice (**Figure 1C**). However, the cellular infiltrate scores obtained from the colon (*p* = 0.0008) and liver (*p* = 0.0036) were higher in Y-infected mice than in DA-infected mice. Similar infiltrate scores were found in the heart and skeletal muscle samples from chronically infected mice with both *T. cruzi* strains (**Figure 1D**). Overall, increased parasitism and inflammation were observed in tissue samples from acutely Y-infected mice, and reduced but identifiable inflammatory scores were observed in the chronically DA-infected mice. Moreover, the acutely and chronically DA-infected mice had lower parasitic and inflammatory outcomes. Thus, our findings indicate that acutely and chronically Y- or DA-infected mice exhibit different outcomes.

### Differential CD8^+^ T Cell Responses Induced by the DA or Y Strain During Acute and Chronic *T. cruzi* Infection

Since distinctive outcomes in mice infected with *T. cruzi* Y or DA strains were observed, we evaluated the relationship between the T cell response in Y- or DA-infected mice and infection outcomes. We examined the CD8^+^ T cell response focused on the following parameters: memory/effector subsets, antigen-specific response, and expression of inhibitory receptors on T cells. Each parameter was defined as described in the Materials and Methods and as shown in **Supplemental Figure 1**.

**Figure 2:**
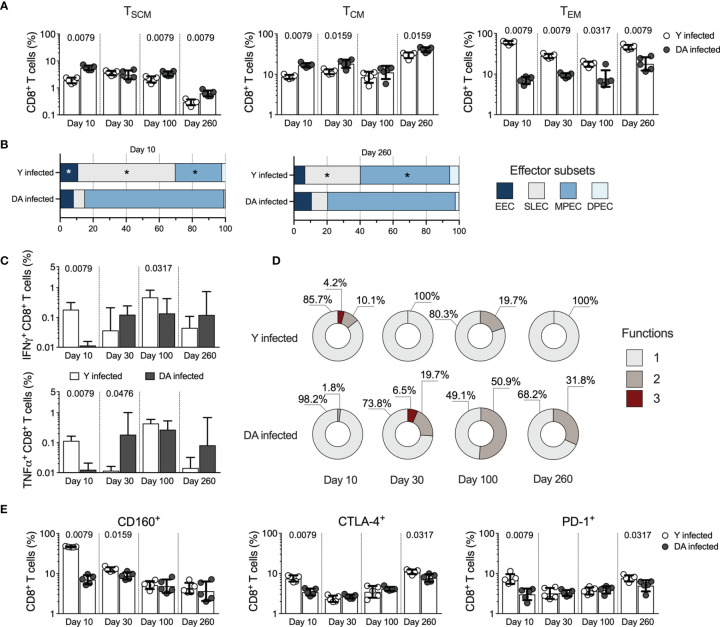
CD8^+^ T cell responses in the acutely and chronically Y- or DA-infected mice. **(A)** Frequency of CD8^+^ T_SCM_, T_CM_, and T_EM_ cells in acutely and chronically Y- or DA-infected mice. **(B)** Proportion of EEC, SLEC, MPEC, and DPEC CD8^+^ T cells in the acutely and chronically Y- or DA-infected mice at 10 and 260 dpi. Significant differences of effector CD8^+^ T cell subsets between Y- and DA-infected mice are shown with an asterisk (*). **(C)** Antigen-specific CD8^+^ T cells producing IFNγ or TNFα in acutely and chronically Y- or DA-infected mice. Background-subtracted data analyzed in all cases. **(D)** Proportion of antigen-specific CD8^+^ T cells with one, two, or three functions, as defined by the production of IFNγ, TNFα, and IL-2 based on a Boolean strategy, in the acutely and chronically Y- or DA-infected mice. **(E)** Frequency of CD8^+^ T cells expressing CD160, CTLA-4, or PD-1 from acutely and chronically Y- or DA-infected mice. The bar graphs in **(A, C, E)** show the geometric mean and geometric SD. Log data analyzed in **(A, C, E)** Each point represents a mouse. Results are pooled from one experiment with five mice per group. The *p* values were calculated using the Mann–Whitney U test. T_SCM_, stem cell memory; T_CM_, central memory; T_EM_, effector memory; EECs, early effector cells; SLECs, short-lived effector cells, MPECs, memory precursor effector cells; DPECs, double-positive effector cells.

The memory CD8^+^ T cell subsets analyzed in the Y- or DA-infected mice included stem cell memory cells (T_SCM_ cells, CD44^-^CD62L^+^CD122^+^CD127^+^), central memory cells (T_CM_, CD44^+^CD62L^+^), and effector memory cells (T_EM_ cells, CD44^+^CD62L^-^). As shown in **Figure 2A**, the Y-infected mice presented reduced percentages of CD8^+^ T_SCM_ (at 10 dpi [*p* = 0.0079], 100 dpi [*p* = 0.0079], and 260 dpi [*p* = 0.0079]) and T_CM_ cells (at 10 dpi [*p* = 0.0079], 30 dpi [*p* = 0.0159], and 260 dpi [*p* = 0.0159]) and increased percentages of CD8^+^ T_EM_ cells (at 10 dpi [*p* = 0.0079], 30 dpi [*p* = 0.0079], 100 dpi [*p* = 0.0317], and 260 dpi [*p* = 0.0079]) compared with the DA-infected mice (**Figure 2A**). These results showed that the Y-infected mice exhibited a memory profile of T cell subsets characterized by a predominant proportion of effector cells consisting of terminally differentiated memory phenotypes (T_EM_ cells), whereas the DA-infected mice exhibited early-differentiated memory phenotypes (T_SCM_ and T_CM_ cells). Because the effector CD8^+^ T cell response in mice can be examined based on KLRG1 and CD127 expression (Joshi et al., 2007; Obar and Sheridan, 2015), we analyzed these effector subsets in Y- or DA-infected mice. The effector CD8^+^ T cell subsets scrutinized in this study included early effector cells (EECs, KLRG1^-^CD127^-^), short-lived effector cells (SLECs, KLRG1^+^CD127^-^), memory precursor effector cells (MPECs, KLRG1^-^CD127^+^), and double-positive effector cells (DPECs, KLRG1^+^CD127^+^) (**Figure 2B**). The results shown in **Figure 2B** indicated that Y- or DA-infected mice showed between the acute and chronic infection stages similar proportions of effector CD8^+^ T cell subsets. Then, we analyzed the effector subsets at 10 or 260 dpi. The obtained data show that Y-infected mice presented high percentages of EECs at 10 dpi and SLEC CD8^+^ T cells at 10 and 260 dpi compared with DA-infected mice. Additionally, the Y-infected mice showed low percentages of MPEC CD8^+^ T cells at 10 and 260 dpi compared with the DA-infected mice (**Figure 2B**). Thus, the Y-infected mice presented short-lived effector cells (EECs and SLECs), whereas the DA-infected mice exhibited memory precursor effector cells (MPECs) in both acutely and chronically infected mice.

To determine whether the functional properties exhibited by antigen-specific T cells are influenced by the *T. cruzi* strain, we subsequently compared the functional antigen-specific CD8^+^ T cells by measuring the production of IFNγ, TNFα, or IL-2 in response to stimulation with soluble antigens from *T. cruzi*. The Y-infected mice exhibited high percentages of IFNγ-producing CD8^+^ T cells at 10 (*p* = 0.0079) and 100 dpi (*p* = 0.0317) and TNFα-producing cells at 10 dpi (*p* = 0.0079) in the Y-infected mice, whereas the DA-infected mice presented a greater percentage of TNFα-producing cells at 30 dpi (*p* = 0.0476) (**Figure 2C**). IL-2 was the cytokine produced at the lowest level in the mice infected with either the Y or DA strain in acute or chronic phases, with no differences in the percentages of IL-2-producing CD8^+^ T cells in either group (**Supplemental Figure 3**). The analysis of the monofunctional and multifunctional responses in *T. cruzi*-specific CD8^+^ T cells showed that Y-infected mice exhibited CD8^+^ T cells with three or two functions at 10 or 100 dpi. In contrast, the DA-infected mice displayed a stronger multifunctional response with three or two functions at 30, 100, or 260 dpi (**Figure 2D**). Overall, Y-infected mice showed monofunctional *T. cruzi*-specific CD8^+^ T cell responses at 30 and 260 dpi, whereas DA-infected mice exhibited monofunctional *T. cruzi*-specific CD8^+^ T cell responses at 10 dpi. Additionally, the multifunctional capacities of Ag-specific CD8^+^ T cells in DA-infected mice were maintained for up to 260 dpi (**Figure 2D**).

To assess the expression of inhibitory receptors on T cells in mice infected with the *T. cruzi* strains, cells from the Y- or DA-infected mice were stained with antibodies against 2B4, CD160, CTLA-4, and PD-1. The frequency of 2B4-expressing CD8^+^ T cells was higher (*p* = 0.0079) at 10 dpi in Y-infected mice (median 4.01% [3.52-4.35]) than in DA-infected mice (median 1.12% [0.98-1.28]). Similar values of 2B4-expressing CD8^+^ T cells were detected in Y- and DA-infected mice at 30, 100, and 260 dpi (Y-infected mice, 1.01%, 1.3%, 1.25%, respectively; DA-infected mice, 1.13%, 1.37%, 0.83%, respectively). CD8^+^ T cells from the Y-infected mice showed increased expression of CD160 (at 10 dpi [*p* = 0.0079] and 30 dpi [*p* = 0.0159]), CTLA-4 (at 10 dpi [*p* = 0.0079] and 260 dpi [*p* = 0.0317]), or PD-1 (at 10 dpi [*p* = 0.0079] and 260 dpi [*p* = 0.0317]) compared with the DA-infected mice (**Figure 2E**). Additionally, CD8^+^ T cells expressing several inhibitory receptors exhibited a greater coexpression profile in the Y-infected mice than in the DA-infected mice at 10 dpi. The predominant profile of coexpression in the chronically infected mice corresponded to CD8^+^ T cells coexpressing CD160, CTLA-4, and PD-1. CD8^+^ T cells from the Y- or DA-infected mice coexpressed similar proportions of inhibitory receptors at 100 and 260 dpi (**Supplemental Figure 4**). Altogether, our results suggest that CD8^+^ T cell responses were strain-specific and differentially modulated during acute and chronic *T. cruzi* infection.

### CD4^+^ T Cell Responses in Y- and DA-Infected Mice in the Acute and Chronic Phases

**Figure 3:**
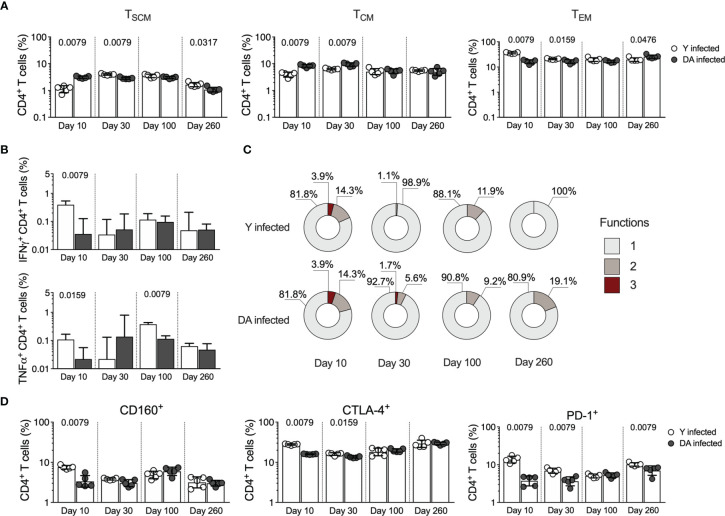
CD4+ T cell responses in acutely and chronically Y- or DA-infected mice. **(A)** Frequency of CD4^+^ T_SCM_, T_CM_, and T_EM_ cells in acutely and chronically Y- or DA-infected mice. **(B)** Antigen-specific CD4^+^ T cells producing IFNγ or TNFα in acutely and chronically Y- or DA-infected mice. Background-subtracted data analyzed in all cases. **(C)** Proportion of antigen-specific CD4^+^ T cells with one, two, or three functions, as defined by the production of IFNγ, TNFα, and IL-2 based on a Boolean strategy, in the acutely and chronically Y- or DA-infected mice. **(D)** Frequency of CD4^+^ T cells expressing CD160, CTLA-4, or PD-1 from acutely and chronically Y- or DA-infected mice. The bar graphs in **(A, B, D)** show the geometric mean and geometric SD. Log data analyzed in **(A, B, D)**. Each point represents a mouse. Results are pooled from one experiment with five mice per group. The *p* values were calculated using the Mann–Whitney U test.

Previous studies have revealed that *T. cruzi* infection promotes the activation of different CD4^+^ T cell profiles involved in activating and regulating immune response processes that may determine the infection outcome (Acevedo et al., 2018). We analyzed the CD4^+^ T cell response in Y- and DA-infected mice, and slight differences were found between the *T. cruzi*-specific CD4^+^ T cell immune responses in Y- and DA-infected mice. Similar to the observed for the CD8^+^ T cell response in Y- and DA-infected mice, the results obtained indicated that chronically DA-infected mice had higher CD4^+^ T_EM_ cell frequencies and increased multifunctional CD4^+^ T cells than Y-infected mice (**Figures 3A–C**), as reported previously in mouse models and humans (Padilla et al., 2007; Albareda et al., 2009; Padilla et al., 2009). The Y-infected mice exhibited high percentages of IFNγ-producing CD4^+^ T cells (at 10 dpi, *p* = 0.0079) and TNFα-producing CD4^+^ T cells (at 10 dpi [*p* = 0.0159] and 100 dpi [*p* = 0.0079]) compared with the DA-infected mice (**Figure 3B**). CD4^+^ T cells from the Y-infected mice showed increased expression of CD160 (at 10 dpi [*p* = 0.0079]), CTLA-4 (at 10 dpi [*p* = 0.0079] and 30 dpi [*p* = 0.0159]), or PD-1 (at 10 dpi [*p* = 0.0079], 30 dpi [*p* = 0.0079] and 260 dpi [*p* = 0.0079]) than those from DA-infected mice (**Figure 3D**). Overall, our results suggest that the CD4^+^ and CD8^+^ T cell responses were strain-specific and differentially modulated during acute and chronic *T. cruzi* infection.

**Figure 4:**
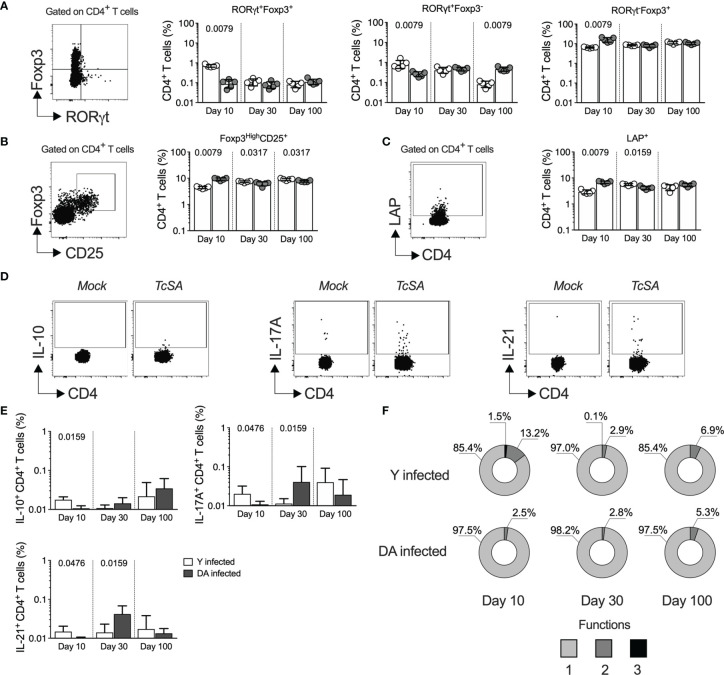
CD4^+^ T helper profiles dissected during early *T. cruzi* infection with two distinct strains. **(A)** Percentages of CD4^+^ T cells showing different expression of RORγt and Foxp3 in the Y- or DA-infected mice. The left panel shows the representative dot plot of the gating strategy for RORγt^+^Foxp3^+^, RORγt^+^Foxp3^-^, or RORγt^-^Foxp3^+^ CD4^+^ T cells. **(B)** Percentages of CD4^+^ T cells coexpressing Foxp3 and CD25 in the Y- or DA-infected mice. The left panel shows the representative dot plot of the gating strategy for expressing Foxp3^High^CD25^+^ CD4^+^ T cells. **(C)** CD4^+^ T cells expressing LAP in the Y- or DA-infected mice. The left panel shows the representative dot plot of the gating strategy for CD4^+^ T cells expressing LAP. **(D)** Representative dot plot of CD4^+^ T cells producing IL-10, IL-17A, or IL-21. The gates applied for the identification of cytokine production among the total population of CD4^+^ T cells were defined according to the cells from each mouse that were cultured with mock. **(E)** Percentages of CD4^+^ T cells producing IL-10, IL-17A, or IL-21 in the Y- or DA-infected mice. **(F)** Proportion of antigen-specific CD4^+^ T cells with one, two, or three functions, as defined by the production of IL-10, IL-17A, and IL-21 based on a Boolean strategy, in the Y- or DA-infected mice. The bar graphs show the geometric meam and geometric SD. Each point represents a mouse. The pie charts show the medians of the CD4^+^ T cells with one, two, or three functions detected in the acutely and chronically Y- or DA-infected mice **(E)**. Results are pooled from one experiment with five mice per group. The *p* values were calculated using the Mann–Whitney U test.

The heterogeneous response of CD4^+^ T cells can be analyzed through the expression of master transcription factors (TBX21, Foxp3, or RORγt) or the profiles of cytokine-producing cells (IFNγ, IL-10, or IL-17A) (Oestreich and Weinmann, 2012). Since an early CD4^+^ T cell response could define *T. cruzi* infection (Guo and Cobb, 2009; Miyazaki et al., 2010; Sanoja et al., 2013; Cai et al., 2016), we next analyzed the CD4^+^ T cell profiles expressing RORγt and Foxp3 transcription factors in Y- and DA-infected mice for up to 100 dpi. As observed in **Figure 4**, at 10 dpi, the Y-infected mice showed a higher percentage (*p* = 0.0079) of double-positive CD4^+^ T cells expressing Foxp3 and RORγt than the DA-infected mice; however, at 30 or 100 dpi, the Y- or DA-infected mice presented similar values of T cells that were positive for both of these transcription factors (**Figure 4A**). Moreover, increased percentages of RORγt^+^Foxp3^-^ cells were detected in the Y-infected mice at 10 dpi (*p* = 0.0079), whereas in the DA-infected mice, the percentages of these cells were increased at 100 dpi (*p* = 0.0079). In addition, the proportions of RORγt^-^Foxp3^+^ cells at 10 dpi were lower in the Y-infected mice than in the DA-infected mice (*p* = 0.0079). Notably, the percentages of CD4^+^ T cells expressing Foxp3 in the mice infected with Y or DA remained similar throughout the other days evaluated in this study (**Figure 4A**). Subsequently, we examined two T regulatory phenotypes, including cells expressing Foxp3 at high levels and CD25, which are known as thymic Tregs (tTregs), and cells expressing latency-associated peptide (LAP), which identifies a population of cells producing TGFβ. Notably, both CD4^+^ T regulatory phenotypes presented similar patterns during acute infection and were dependent on the *T. cruzi* strain because Foxp3^Hi^CD25^+^ and LAP^+^ cells were significantly increased in the DA-infected mice at 10 dpi (*p* = 0.0079) and were found at higher proportions in the Y-infected mice at 30 dpi (*p* = 0.0317 and *p* = 0.0159) (**Figure 4B**). In addition, the percentages of CD4^+^ T cells expressing Foxp3^Hi^CD25^+^ were also increased at 100 dpi in the Y-infected mice compared with the DA-infected mice (*p* = 0.0317) (**Figure 4C**). We subsequently identified the CD4^+^ T cell profiles by measuring the CD4^+^ T cells that produce IL-10, IL-17A, or IL-21 in the Y- or DA-infected mice (**Figure 4D**). The Y-infected mice showed increased percentages of CD4^+^ T cells producing IL-10 (*p* = 0.0159), IL-17A (*p* = 0.0476), or IL-21 (*p* = 0.0476) at 10 dpi, whereas the DA-infected mice exhibited high frequencies of cells producing IL-17A (*p* = 0.0159) or IL-21 (*p* = 0.0159) at 30 dpi. Similar values of cytokine-producing CD4^+^ T cells were observed between the Y- and DA-infected mice at 100 dpi (**Figure 4E**). The analysis of the proportions of CD4^+^ multifunctional/monofunctional responses based on measurements of the above-mentioned cytokines at 10 dpi revealed greater amounts of IL-10^+^IL-17A^+^IL-21^+^ cells in the Y-infected mice than in the DA-infected mice; however, the Y- and DA-infected mice exhibited similar monofunctional and multifunctional patterns of CD4^+^ T cell responses after stimulation with soluble antigens from *T. cruzi* at 30 and 100 dpi (**Figure 4F**).

Overall, our results suggest that differences in the infection outcomes at the acute and chronic stages after infection with genetically different *T. cruzi* strains might be dictated by CD8^+^ and CD4^+^ T cell responses, including early effector and regulatory mechanisms. Nevertheless, it is unknown whether these differences are maintained in a mixed chronic infection by different *T. cruzi* DTUs.

### Different Outcomes of Chronic Homologous and Heterologous *T. cruzi* Infection in Mice

**Figure 5:**
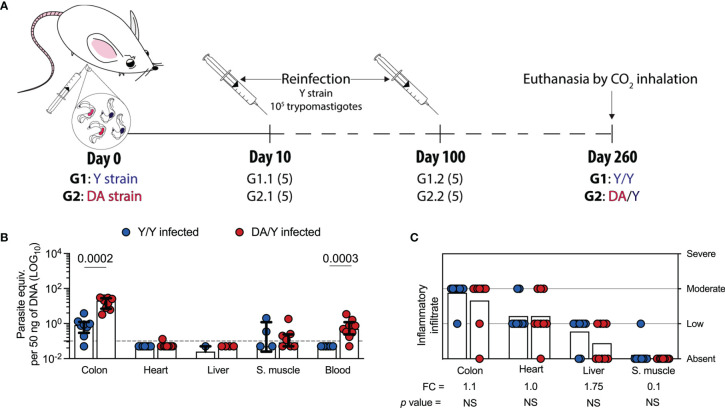
Parasite loads and inflammatory infiltrate scores of tissues from Y/Y- or DA/Y-infected mice. **(A)** Schematic representation of the reinfection of mice with *T. cruzi*. The mice were infected with either the Y or DA strain, reinfected with the Y strain at 10 or 100 dpi, and euthanized by CO_2_ inhalation at 260 dpi. The mice in group 1 (G1) were infected and reinfected with the Y strain (Y/Y), and those in group 2 (G2) were infected with DA and reinfected with the Y strain (DA/Y). The mice reinfected at 10 or 100 dpi were grouped into the Y/Y or DA/Y groups. Numbers in parentheses represent the number of mice included in each group. **(B)** Parasite loads in colon, heart, liver, skeletal muscle, and blood samples from the Y/Y- or DA/Y-infected mice. The bar graphs show the medians and ranges of the parasite equivalent per 50 ng of DNA (LOG_10_) in tissues from each group of challenged mice. The dotted line represents the cutoff for the LOQ based on serially diluted *T. cruzi*-spiked tissue DNA as described in the Materials and Methods. **(C)** Inflammatory infiltrate scores obtained for colon, heart, liver, and skeletal muscle samples from the Y/Y- or DA/Y-infected mice. The bar graphs show the average inflammatory infiltrate scores in the tissues from each group of challenged mice. FC was determined as the inflammatory score detected in the Y/Y mice divided by the infiltrate inflammatory score found in the DA/Y mice. Log data analyzed in **(B)** Each point represents the value of the parasite load and infiltrate inflammatory score detected in each mouse under the above-described conditions. Results are pooled from one experiment with nine mice per group. The *p* values were calculated using the Mann–Whitney U test.

Because several studies have demonstrated that the *T. cruzi* genotypes and reinfections could define the outcome of infection in humans and animal models (Basombrio and Besuschio, 1982; Basombrio et al., 1982; Bustamante et al., 2002; Bustamante et al., 2007), we examined the parasitological and histological outcomes after homologous or heterologous infection by *T. cruzi* strains in a chronic setup. Since we characterized the outcomes and T cell response in a set of single infection experiments with Y or DA, we next evaluated the outcomes in homologous and heterologous infection with *T. cruzi* strains (Y/Y or DA/Y). Thus, mice were first infected with the Y (G1) or DA (G2) strain, challenged with the Y strain at 10 or 100 dpi, and euthanized at 260 dpi **(** **Figure 5A** **)**. Since the G1- and G2-reinfected groups at 10 or 100 dpi showed similar parasitological and histological outcomes at 260 dpi, both groups were organized as single groups in the subsequent analyses. They were named as Y/Y or DA/Y groups, respectively. Colon (*p* = 0.0002) and blood (*p* = 0.0003) samples from the Y/Y-infected mice showed lower parasite loads than tissue samples from the DA/Y-infected mice. The parasite loads in heart and skeletal muscle tissues from the Y/Y-infected mice were similar to those from the DA/Y-infected mice **(** **Figure 5B** **)**. Although no differences in the parasite load were detected in liver tissue samples from the Y/Y and DA/Y groups, a higher number of liver tissue samples from the DA/Y-infected mice had detectable levels of *T. cruzi*. In addition, the colon, heart, liver, and skeletal muscle tissue samples from both reinfected groups showed similar inflammatory scores (**Figure 5C**). Thus, in contrast to the tissue parasite burden, the inflammatory infiltrate score was not a differential parameter of the infection outcome. They exhibited similarities during chronic homologous and heterologous reinfections with *T. cruzi* Y- and DA- strains.

### Homologous and Heterologous Infection of Mice With *T. cruzi* Strains Showed a Contrasting T Cell Response

**Figure 6:**
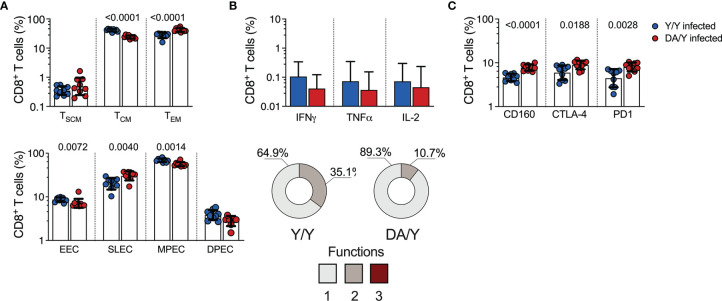
CD8^+^ T cell responses in the Y/Y- or DA/Y-infected mice. **(A)** Frequency of memory (top panel) or effector (bottom panel) CD8^+^ T cell subsets in the Y/Y- or DA/Y-infected mice. **(B)** Antigen-specific CD8^+^ T cells producing IFNγ, TNFα, or IL-2 in the Y/Y- or DA/Y-infected mice. The bottom pie charts show the median of the CD8^+^ T cells with one, two, or three functions in the Y/Y- or DA/Y-infected mice. **(C)** Frequency of CD8^+^ T cells expressing inhibitory receptors in the Y/Y- or DA/Y-infected mice. The bar graphs in **(A–C)** show the geometric mean and geometric SD. Log data analyzed in **(A–C)**. Each point represents a mouse. Results are pooled from one experiment with nice mice per group. The *p* values were calculated using the Mann–Whitney U test. T_SCM_, stem cell memory; T_CM_, central memory; T_EM_, effector memory; EECs, early effector cells; SLECs, short-lived effector cells, MPECs, memory precursor effector cells; DPECs, double-positive effector cells.

To investigate whether our observations in mice after mixed sequential infection with *T. cruzi* strains are linked to the T cell response quality, we assessed the memory and effector subsets, antigen-specific T cell populations that produce various cytokines, and the expression of inhibitory receptors on T cells as described above. The comparison of the CD8^+^ T cell memory distribution in mice with homologous or heterologous infection revealed that the Y/Y-infected mice showed higher percentages of CD8^+^ T_CM_ cells and lower percentages of T_EM_ cells than the DA/Y-infected mice. The Y/Y- and DA/Y-infected mice displayed similar percentages of CD8^+^ T_SCM_ cells. Additionally, the Y/Y-infected mice exhibited higher EEC and MPEC CD8^+^ T cells and lower SLEC CD8^+^ T cells than the DA/Y mice **(** **Figure 6A** **)**. The antigen-specific CD8^+^ T cells producing cytokines in homologous or heterologous reinfected mice were then analyzed. The Y/Y- and DA/Y-infected mice showed similar percentages of IFNγ-, TNFα-, and IL-2-producing CD8^+^ T cells. In addition, the Y/Y-infected mice presented a higher proportion of multifunctional CD8^+^ T cells with two functions than the DA/Y-infected mice (**Figure 6B**). Further examination of the individual expression of inhibitory receptors on CD8^+^ T cells showed that the Y/Y-infected mice exhibited lower frequencies of CD160, CTLA-4, or PD-1 than the DA/Y group (**Figure 6C**). However, the Y/Y- and DA/Y-infected mice exhibited a similar proportion of CD8^+^ T cells coexpressing inhibitory receptors (**Supplemental Figure 5**).

**Figure 7:**
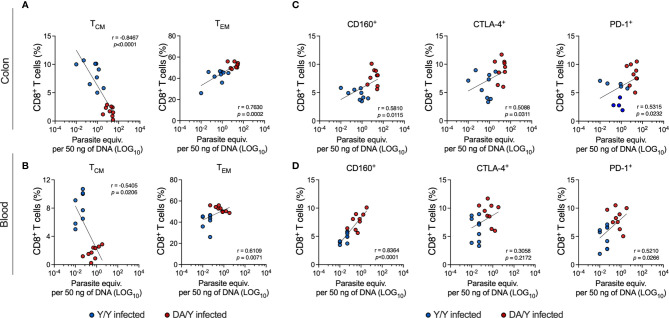
Correlation analysis between CD8^+^ T cell responses and tissue parasite loads in the Y/Y- and DA/Y-infected mice. **(A)** Correlation analysis of the parasite loads in colon samples from the Y/Y- and DA/Y-infected mice with the percentages of CD8^+^ T_CM_ and T_EM_ cells. **(B)** Correlation analysis of the parasite loads in blood samples from the Y/Y- and DA/Y-infected mice with the percentages of CD8^+^ T_CM_ and T_EM_ cells. **(C)** Correlation analysis of the parasite loads in colon samples from the Y/Y- and DA/Y-infected mice with the percentages of CD8^+^ T cells expressing inhibitory receptors. **(D)** Correlation analysis of the parasite loads in blood samples from the Y/Y- and DA/Y-infected mice with the percentages of CD8^+^ T cells expressing inhibitory receptors. The *r* and *p* values were calculated using the Spearman rank test.

Thus, we subsequently examined the relationship between parasite control and the T cell response in reinfected mice in a chronic setup. A Spearman correlation analysis was performed between the parasite load detected in colon or blood samples and the percentages of memory T cell subsets or T cells expressing inhibitory receptors. We correlated the readouts differently arranged in reinfected Y/Y and DA/Y mice (data shown in **Figures 5**, **6**). The analysis of the correlations in colon and blood samples showed similar patterns with slight differences. Parasite loads detected in the colon and blood samples were inversely correlated with the percentages of CD8^+^ T_CM_ cells (r = -0.8467, *p* < 0.0001; r = 0.5405, *p* = 0.0206, respectively). Additionally, the parasite loads in the colon and blood samples were positively correlated with the percentages of CD8^+^ T_EM_ cells (r = 0.7630, *p* = 0.0002; r = 0.6109, *p* = 0.0071, respectively) and the percentages of CD8^+^ T cells expressing CD160 (r = 0.5810, *p* = 0.0115; r = 0.8364, *p* < 0.0001, respectively), CTLA-4 (r = 0.5088, *p* = 0.0311; r = 0.3058, *p* = 0.2172, respectively), and PD-1 (r = 0.5315, *p* = 0.0232; r = 0.5210, *p =* 0.0266, respectively) (**Figure 7**). Overall, these results show that homologous infections by *T. cruzi* show lower parasitic load, while *T. cruzi* heterologous infections present a greater parasitic load. Interestingly, these two scenarios allowed us to establish correlations between the result of the infection measured by parasite loads and the response of T cells. These findings suggest that in the presence of high parasitic loads given by mixed infections of different genotype strains, there is an increased presence of effector T cells and higher expression of inhibitory receptors. Notwithstanding, lower parasite loads in *T. cruzi* -infected mice are related to a predominance of early-differentiated T cells and lower expression of inhibitory receptors, as observed in the homologous Y/Y group. This study provides evidence that the immune T cell response correlates with parasitic outcomes during chronic *T. cruzi* infection.

In summary, the two different approaches used in this study detected contrasting parasitological and histological outcomes accompanied by a differential T cell response in mice inoculated with *T. cruzi* strains belonging to two different genotypes (Y versus DA) and in mice that were homologously or heterologously reinfected with the *T. cruzi* strains (Y/Y versus DA/Y). The evaluation of the T cell response in chronically infected mice with a low inflammatory infiltrate (DA-infected mice) or low parasitemia and parasitism (Y/Y-infected mice) revealed an increase in early-differentiated T cells, a multifunctional T cell response and lower expression of inhibitory receptors on T cells. In contrast, in chronically infected mice with a high inflammatory infiltrate (Y-infected mice) or high parasitemia and parasitism (DA/Y-infected mice), the T cell response was distinguished by an increase in late-differentiated cells, a monofunctional response, and an increment in the expression of inhibitory receptors by T cells. Collectively, this study demonstrates that the quality of the T cell response is related to parasite control during chronic *T. cruzi* infection **(** **Table 1** **)**.

**Table 1:** Infection outcomes and T cell responses detected in chronically *T. cruzi*-infected or reinfected mice.

	Infected		Reinfected	
	Y	DA	Y/Y	DA/Y
***Infection outcome***				
Parasite load^†^	→←	→←	↓	↑
Inflammatory infiltrate	↑	↓	→←	→←
***T cell subsets***				
Early-differentiated (T_CM_)^†^	↓	↑	↑	↓
Late-differentiated (T_EM_)^†^	↑	↓	↓	↑
***Functional activity***				
Profile of the response	One function	Two functions	Two functions	One function
***Inhibitory receptors***				
Individual expression^†^	↑	↓	↓	↑

^†^These variables were positively or negatively correlated in T. cruzi-reinfected mice.

Increases or decreases in these parameters related to the infection outcome or the T cell response are represented with upward or downward arrowheads, respectively. Additionally, the results that were similar between the infected or reinfected groups of mice are depicted as.

## Discussion

Parameters of the quality of the T cell immune response have been evaluated in viral, bacterial, fungal, and protozoan infections. One of the most studied infection models is that caused by lymphocytic choriomeningitis virus (LCMV), and several investigations have revealed differential patterns of immune quality during acute and chronic infection (Wherry et al., 2003; Wherry et al., 2007; Appay et al., 2008). The two scenarios are as follows: i) acute infection induces a potent and protective response that results in clearance of the pathogen and is characterized by early-differentiated antigen-specific T cells with a high functional profile, whereas ii) chronic infection leads to a lethal outcome accompanied by an uncontrollable viral burden in tissues, late-differentiated antigen-experienced T cells, reduced functional T cell capacities, and increased expression of inhibitory receptors on T cells. Comparable findings have been obtained using HIV, tuberculosis, leishmania, toxoplasma, and other infectious disease models (Betts et al., 2006; Gigley et al., 2012; Singh et al., 2013; McLane et al., 2019). Here, we investigated whether the parasitological or histological outcomes are related to the quality of the T cell response during chronic *T. cruzi* infection, which is a neglected infectious disease that primarily affects underprivileged populations and for which there is no vaccine or treatment available. Although many studies have suggested that ChD progression is due to deterioration in the quality of the immune response, a direct relationship between T cell responses and disease outcome has not been established. Defining the outcome of the disease has been difficult because 30-40% of individuals exhibit mainly cardiac or digestive complications decades after the initial infection. Thus, we attempted to determine the histological and parasitological outcomes and dissected the T cell responses in mice infected with two distinct *T. cruzi* strains or subsequently challenged with the parasite to identify the relationship between parasite control and the immune response.

First, a set of single infection experiments was performed using two *T. cruzi* strains, Y (TcII) and DA (TcI), and the parasite loads and the inflammatory infiltrate scores of tissues and the T cell responses were then compared between the infected groups at the acute (at 10 and 30 dpi) and chronic stages (at 100 and 260 dpi). In mice, infection with either Y or DA *T. cruzi* led to contrasting outcomes and T cell responses. Similar to the results from a previous report conducted by our group (Mateus et al., 2019), acute Y infection induced high antigen-specific multifunctional CD8^+^ T cells by producing IFNγ, TNFα and IL-2 and high percentages of T cells coexpressing 2B4, CD160, CTLA-4, and PD-1. Chronically Y-infected mice exhibited a moderate inflammatory infiltrate in colon and liver tissues accompanied by poor T cell effector function and increased coexpression of inhibitory receptors by CD8^+^ T cells. Interestingly, the DA-infected mice showed reduced or undetectable tissue parasite loads, a reduced tissue inflammatory infiltrate, a multifunctional T cell response detectable at 30, 100, and 260 dpi and reduced expression or coexpression of inhibitory receptors. Several studies support that different *T. cruzi* genotypes can infect different tissues in humans and in experimentally infected murine models are able to induce different degrees of damage and therefore drive distinct infection outcomes. These differences have been associated with several biological features of the parasite, including dormancy/reactivation, persistence, low-level infection, tissue tropism, and episodic reinvasion (Lewis and Kelly, 2016; Sanchez-Valdez et al., 2018; Zingales, 2018). We and other research groups have described the histological and parasitological characteristics of Y infection in mouse models (Pinto et al., 1999; Sanoja et al., 2013; Mateus et al., 2019). Certainly, our findings regarding the inflammatory infiltrate, parasite loads, and T cell responses measured in the acutely and chronically Y-infected mice were quite consistent with those detected in a previous study (Mateus et al., 2019) and suggested that *T. cruzi* persistence could promote the dysfunctionality of T cells, similar to the results obtained for several chronic infectious diseases in humans and murine models. In contrast, only a few studies have evaluated the outcomes in animal models infected with the DA strain (Barrera et al., 2008; Cruz et al., 2015), and these investigations showed that the genotype of the DA strain (i.e., TcI) tends to be detectable in blood and can invade tissues (Cruz et al., 2015), as described in Colombian patients infected with TcI strains (Ramirez et al., 2010). In this study, the parasite was detected in tissue as described previously (Barrera et al., 2008; Cruz et al., 2015), but lower parasitic loads were measured in the samples from the DA-infected mice than in those from the Y-infected mice. We hypothesized that this difference might be related to the DTU and the degree of virulence of the strain. In this sense, the reduced tissue parasite burden and the lower inflammatory infiltrate score detected in the DA-infected group might be a consequence of the strong T cell response detected in this group. Indeed, this hypothesis is supported because several studies have suggested that a multifunctional CD8^+^ T cell response after vaccination might be related to controlled *T. cruzi* infection (de Alencar et al., 2009; Vasconcelos et al., 2014), which was similar to the findings observed in DA-infected mice. Thus, this study underlines two different results regarding the relationship between the infection outcome and the T cell response and suggests that the immune T cell response detected in Y- or DA-infected mice is associated with the differential outcomes detected in *T. cruzi*-infected mice. Altogether, this work ratifies that the study of strains of *T. cruzi* isolated from infected individuals can offer an opportunity to explore the differences detected in chronically *T. cruzi*-infected individuals and the heterogeneity of the clinical outcomes observed in chagasic patients.

We analyzed additional profiles of CD4^+^ T cells in Y- and DA-infected mice to determine whether the early responses of CD4^+^ T cells contribute to the differential responses detected. Y infection leads to increased regulatory (IL-10-producing CD4^+^ T cells and Foxp3^+^RORγt^+^ cells) and effector profiles (IL-17-, IL-21-producing CD4^+^ T cells and RORγt^+^ cells) during the early acute phase (10 dpi), whereas delayed activation of these cellular mechanisms was detected in the DA-infected mice at 30 dpi. Additionally, the Y-infected mice showed additional regulatory mechanisms (Foxp3^Hi^CD25^+^CD4^+^ T cells and LAP^+^CD4^+^ T cells) during the late stage of acute infection and tended to increase during early chronic infection. This finding suggests that induction of the regulatory response during acute *T. cruzi* infection limits infection control to avoid damage. In contrast, the induction of a regulatory response during chronic infection can promote deterioration of the T cell response, leading to an uncontrolled infection, as previously demonstrated (Waghabi et al., 2005; Guedes et al., 2012). It is important to highlight that this study constitutes the first investigation that compares other CD4 profiles through a set of single-infection experiments with two *T. cruzi* strains that yield differential outcomes. Previous reports highlight the importance of CD4^+^ T cell functional subsets during *T. cruzi* infection, such as Th17 and Treg cells (Guo and Cobb, 2009; Miyazaki et al., 2010; Sanoja et al., 2013; Cai et al., 2016). However, according to several findings, these cell populations might be related to both susceptibility and protection against acute or chronic *T. cruzi* infection. For instance, IL-17 is crucial for the control of acute infection and helps the differentiation of Th1 cells in infected mice (da Matta Guedes et al., 2010; Miyazaki et al., 2010). In contrast, some studies have shown that IL-17 production is related to susceptibility to acute and chronic *T. cruzi* infection (Vitelli-Avelar et al., 2005; Monteiro et al., 2007). Additionally, CD4^+^ Th17 cells can provide critical contributions to the CD8^+^ T cell response induced by the secretion of IL-21, which is a cytokine related to the Th17 phenotype and T follicular helper cells (Cai et al., 2016). Indeed, we evaluated the population of IL-21-producing CD4^+^ T cells in response to *T. cruzi* soluble antigens and observed similar patterns of IL-17 and IL-21 production in the Y- and DA-infected mice, which suggested that both cytokines are produced by Th17 cells. However, whether the production of IL-21 detected in *T. cruzi*-infected mice also aids the induction of T follicular helper cells and thus the activation of B cells and the production of antibodies against *T. cruzi* antigens remains unclear (Liu and King, 2013; Leonard and Wan, 2016). Of note, a multifunctional effector/regulatory response dictated by IL-10^+^IL-17^+^IL-21^+^ cells and increased percentages of Foxp3^+^ T cells expressing RORγt was detected in the Y-infected mice at 10 dpi. Although unconventional, this phenotype of CD4^+^ T cells has been detected in infection models and autoimmune diseases with potently suppressive capacities in mucosal inflammation (Littman and Rudensky, 2010; Solomon and Hsieh, 2016; Yang et al., 2016). Based on these findings, we hypothesize that these populations of CD4^+^ T cells can potently regulate the effector response in Y-infected mice.

In addition, previous studies have shown that IL-10-producing CD4^+^ T cells, Foxp3^Hi^CD25^+^ cells and LAP^+^ CD4^+^ T cells play a crucial role during acute and chronic *T. cruzi* infection (Mariano et al., 2008; de Araujo et al., 2011; Bonney et al., 2015; Ferreira et al., 2016; Ferreira et al., 2019). Our results suggest the occurrence of different regulatory mechanisms depending on the infection stage because the Y-infected mice displayed higher percentages of IL-10-producing cells at 10 dpi and increased percentages of Foxp3^Hi^CD25^+^ and LAP^+^ cells at 30 dpi. These results support our hypothesis that the regulatory mechanisms might be a key factor at the acute stage because the immune regulatory response in the Y-infected mice at 30 dpi, as determined by Foxp3^Hi^CD25^+^ and LAP^+^ cells, controls the effector mechanisms observed during the early stage of acute infection. Indeed, previous studies have shown that the high percentages of Foxp3^+^CD4^+^ T cells during acute *T. cruzi* infection might determine the induction of protective CD8^+^ T cell responses (Ersching et al., 2016; Araujo Furlan et al., 2018). Consequently, we hypothesize that effector and regulatory mechanisms are related to the outcomes after infections with the different strains for the following reasons: higher but transitory effector and regulatory mechanisms were detected in the Y-infected mice, whereas the effector and regulatory responses appeared to be delayed but effective in the DA-infected mice because the DA-infected mice showed a no severe outcome during acute and chronic *T. cruzi* infection, as described above. However, the role of these populations during late chronic Y or DA infections needs to be assessed in detail.

Epidemiological studies of individuals with positive Chagas serological tests have shown that continuous exposure to repeated infections increases the risk of progression to chronic Chagas cardiomyopathy (Zicker et al., 1990; Basquiera et al., 2003; Sabino et al., 2013; Perez et al., 2014) and suggest that mixed infection challenges are feasible in naturally infected individuals. Additionally, several studies have evaluated the effect of reinfections in an animal model of *T. cruzi* infection. For example, mixed *T. cruzi* infection can lead to a disseminated infection, electrocardiographic abnormalities and significant increases in parasite loads and tissue fibrosis (Bustamante et al., 2002; Bustamante et al., 2007; Perez et al., 2018; Weaver et al., 2019). Nonetheless, not all infections and reinfections have the same consequences in the host, and because the biological interactions among the *T. cruzi* strains influence the infection outcomes (Ragone et al., 2015; Strauss et al., 2018), we hypothesize that this scenario might be related to the genetic parasite heterogeneity and the host. In this study, we analyzed the outcomes and the quality of the T cell immune response in mice with homologous (Y/Y) or heterologous (DA/Y) reinfection. The colon and blood samples from the Y/Y-infected mice showed reduced parasite loads than those from the DA/Y-infected mice. Previous reports suggest a positive correlation between the inflammatory infiltrate and the deterioration of cardiac function in infected and reinfected mice (Guerreiro et al., 2015). Although we also sought to establish a relationship between the parasite load and inflammatory infiltrate in tissue samples from Y/Y- or DA/Y-infected mice, similar inflammatory infiltrate scores were found in the tissue samples from both groups. This exciting hypothesis should be confirmed through further studies using deep methods for assessing the pathology of murine tissues showing distinct parasite loads and different inflammatory infiltrates, as previously done (Santi-Rocca et al., 2017; Weaver et al., 2019). In addition, although the Y/Y- and DA/Y-infected mice showed contrasting parasite loads in the colon and blood and these loads were inversely correlated with the percentages of T_CM_ cells or positively correlated with the percentages of T_EM_ cells and the expression of CD160, PD-1, and CTLA-4 on T cells, similar functional activity was found between homologous and heterologous reinfected mice; hence, these parameters were not correlated with the parasite load in the tissue samples collected. However, the T cell functional response is related to the control of parasite infection in mice with drug-cured experimental *T. cruzi* infections and reinfections, as recently described (Mann et al., 2020).

This work lacks cardiac function assessment, which would offer a complete picture of chronic *T. cruzi* infection and reinfection consequences. Although the analysis of the T cell response using strain-specific *T. cruzi* proteins could help to define whether the T cell responses detected can be influenced by the parasite-derived proteins used, it will be important to demonstrate the direct relationship between the parasite load and cardiac function. Additionally, monitoring the inoculated mice for an extended period of time would allow us to define whether the T cell response in DA-infected or Y/Y-reinfected mice is maintained for a longer period than 260 days. Here, it was carried out each experiment including five to nine biological replicates per group. Each experiment was performed to analyze the single and mixed *T. cruzi* infections included mice followed up to 260 days. Our results in *T. cruzi*-infected mice using single or mixed infections demonstrated that *T. cruzi* strains of two different genotypes could affect the infection outcomes and the T cell immune responses induced by the infections, as discussed above. Additional studies are needed to explore the heterogeneity of the *T. cruzi* response-induced in animal models using enough experimental replicates to explore the course and outcome of individual and mixed infections with several *T. cruzi* genetic groups.

This study provides the first demonstration that the immune T cell response correlates with parasitic outcomes during chronic *T. cruzi* infection. Moreover, this evidence supports that the progression of *T. cruzi* infection is related to a decline in the immune T cell response and parasite persistence. This above mentioned is supported by evidence of the scientific literature, as follows: i) chagasic patients coinfected with HIV/AIDS with low CD4^+^ T cell counts exhibited reactivation of *T. cruzi* infection, which results in high mortality due to severe complications in the central nervous system and myocardium (Martins-Melo et al., 2012; Guidetto et al., 2019); ii) an increased number of *T. cruzi*-infected individuals under immunosuppressive therapy present reactivation of the infection with detectable parasitemia in the blood (Campos et al., 2008; da Costa et al., 2017); iii) repeated infections increase the risk of progression to the symptomatic stage in developing cardiomyopathy (Bustamante et al., 2002; Basquiera et al., 2003; Bustamante et al., 2007); and iv), the administration of therapy based on antiparasitic drugs to asymptomatic patients with ChD prevents the occurrence of electrocardiographic alterations (Fragata-Filho et al., 2016). However, the direct relationship between the immune response and parasite persistence has not been established. Thus, we demonstrated that the infection outcomes caused by single or mixed *T. cruzi* infection with different genotypes induce a differential immune T cell response quality. These findings suggest that differences in the infection outcomes at the chronic *T. cruzi* stage after infection or reinfection with genetically different *T. cruzi* strains might be dictated by CD8^+^ T cell responses. We conclude that the CD8^+^ T cell response quality is related to parasite control in chronic *T. cruzi* infection, which supports the notion that parasite persistence and deterioration of the immune T cell response are related to ChD pathology. Monitoring the immunological responses could improve our understanding of *T. cruzi* infection and the development of new therapeutic strategies.

## Data Availability Statement

The original contributions presented in the study are included in the article/**Supplementary Material**. Further inquiries can be directed to the corresponding authors.

## Ethics Statement

This study was performed in accordance with the ethical standards provided by the Institutional Animal Care and Use Committee (IACUC, approval FUA-007-14) of the Unidad de Biología Comparativa (UBA) at Pontificia Universidad Javeriana. All animal studies and protocols were conducted in accordance with the “Guide for the Care and Use of Laboratory Animals” from UBA. Additionally, this study was approved by the Research and Ethics Committees of the Facultad de Ciencias at Pontificia Universidad Javeriana.

## Author Contributions

Conceptualization: JM, PL, ML, CP, and AC. Formal analysis: JM, JG, CP, and AC. Funding acquisition: JM, PL, CP, and AC. Methodology: JM, PN, and CC. Writing – first draft: JM. Writing – review & editing: JM, PN, PL, ML, MT, AE, CC, JG, CP, and AC. All authors contributed to the article and approved the submitted version.

## Funding

JM was supported by Ph.D. scholarships from the Departamento Administrativo de Ciencia, Tecnología e Innovación (COLCIENCIAS) and PUJ (Convocatoria 727 Doctorados Nacionales). ML and MT were supported by grant SAF2016-80998-R from Programa Estatal I+D+I (MINECO, Spain). This work was supported by grants from COLCIENCIAS (code: 120365842534, contract no. FP44842- 615-2014) and the PUJ (proposal ID 6233 and proposal ID 7677).

## Conflict of Interest

The authors declare that the research was conducted in the absence of any commercial or financial relationships that could be construed as a potential conflict of interest.

## Publisher’s Note

All claims expressed in this article are solely those of the authors and do not necessarily represent those of their affiliated organizations, or those of the publisher, the editors and the reviewers. Any product that may be evaluated in this article, or claim that may be made by its manufacturer, is not guaranteed or endorsed by the publisher.
