# Supplementary material for: CD8+ T Cell Response Quality Is Related to Parasite Control in an Animal Model of Single and Mixed Chronic Trypanosoma cruzi Infections
Source: Front Cell Infect Microbiol. 2021 Oct 12;11:723121. doi: 10.3389/fcimb.2021.723121 (PMC8546172; doi:10.3389/fcimb.2021.723121)
Supplement: Supplementary file 1 [file DataSheet_1.docx]

**CD8^+^ T cell response quality is related to parasite control in an animal model of single and mixed chronic *Trypanosoma cruzi* infections**

Jose Mateus^1*^, Paola Nocua^1^, Paola Lasso^2^, Manuel Carlos López^3^, M. Carmen Thomas^3^, Adriana Egui^3^, Claudia Cuervo^1^, John Mario González^4^, Concepción J. Puerta^1^, Adriana Cuéllar^5*^

^1^Grupo de Enfermedades Infecciosas, Facultad de Ciencias, Pontificia Universidad Javeriana, Bogotá, Colombia.

^2^Grupo de Inmunobiología y Biología Celular, Facultad de Ciencias, Pontificia Universidad Javeriana, Bogotá, Colombia.

^3^Instituto de Parasitología y Biomedicina López Neyra, Consejo Superior de Investigaciones Científicas, Granada, Spain.

^4^Grupo de Ciencias Básicas Médicas, Facultad de Medicina, Universidad de los Andes, Bogotá, Colombia.

^5^Grupo de Ciencias de Laboratorio Clínico, Facultad de Ciencias, Pontificia Universidad Javeriana, Bogotá, Colombia.

Corresponding author: [jmtrivino@lji.org](mailto:jmtrivino@lji.org) (J.M.); [acuellar@javeriana.edu.co](mailto:acuellar@javeriana.edu.co) (A.C.)

Supplementary Material

**Supplemental Figure 1. Description of the gating strategy for CD4^+^ and CD8^+^ T cells.** (A) Lymphocytes were first identified in forward scatter (FSC) and side scatter (SSC) plots. The doublets were excluded based on the FSC-A vs. FSC-H and SSC-H vs. SSC-W plots. The dead cells were then excluded from the analysis, and the CD3^+^ and CD4^+^ or CD8^+^ population was selected. (B) Representative dot plot of the gating strategy for memory CD4^+^ or CD8^+^ T cell subsets. Each phenotype was discriminated as follows: stem cell memory (T_SCM_, CD62L^+^CD44^-^CD122^+^CD127^+^), central memory (T_CM_, CD62L^+^CD44^+^), effector memory (T_EM_, CD62L^-^CD44^+^) double-negative (T_DN_, CD62L^-^CD44^-^) cells. The effector CD8^+^ T cell phenotypes were discriminated as follows: early effector cells (EECs, KLRG1^-^CD127^-^), short-lived effector cells (SLECs, KLRG1^+^CD127^-^), memory precursor effector cells (MPECs, KLRG1^-^CD127^+^), and double-positive effector cells (DPECs, KLRG1^-^CD127^+^). (C) Gating strategy for IFNγ-, TNFα-, or IL-2-producing CD4^+^ or CD8^+^ T cells after stimulation with mock or TcSA. The gates applied for the identification of cytokine production among each total population of T cells were defined according to the cells from each mouse that were cultured with mock. (D) Representative dot plot of the gating strategy for CD4^+^ or CD8^+^ T cells expressing 2B4, CD160, CTLA-4, or PD-1.

**
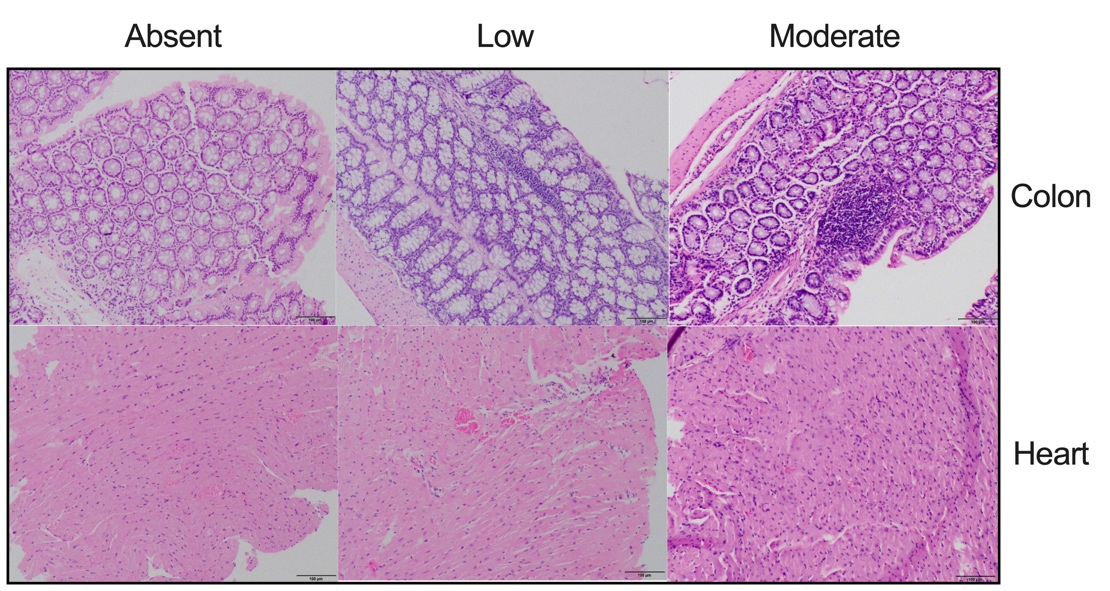
**

**Supplemental Figure 2**. Representative histopathological staining pictures of cross-sections of sample tissues from *T. cruzi*-infected mice.

**Supplemental Figure 3. IL-2-producing CD8^+^ T cell responses in the acutely and chronically Y- or DA-infected mice.** Antigen-specific CD8^+^ T cells producing IL-2 in acutely and chronically Y- or DA-infected mice. Background-subtracted and log data analyzed in all cases. The bar graphs show the geometric mean and geometric SD. Results are pooled from one experiment with five mice per group.

**Supplemental Figure 4.** **Proportion of CD8^+^ T cells coexpressing inhibitory receptors from acutely and chronically Y- or DA-infected mice.** The pie charts show the medians of the CD8^+^ T cells expressing one, two, or more than three inhibitory receptors detected in the acutely and chronically Y- or DA-infected mice. Increasing or decreasing proportions of CD8^+^ T cells that coexpress inhibitory receptors are indicated with upward or downward arrowheads, respectively. The *p* values were calculated using the Mann–Whitney U test; **p*<0.05, ***p*<0.01, ****p*<0.001, *****p*<0.0001.

**Supplemental Figure 5.** **Proportion of CD8^+^ T cells coexpressing inhibitory receptors from Y/Y- or DA/Y-infected mice.** The bar graphs show the geometric mean and geometric SD. Log data analyzed in all cases. Results are pooled from one experiment with nine mice per group.

**Supplemental Table 1. List of antibodies used in the present study.**

| **Marker** | **Fluorochrome** | **Clone** | **Manufacturer** |
| --- | --- | --- | --- |
| CD3 | PerCP-Cy5.5 | 17A2 | BD Biosciences |
| CD4 | Alexa Fluor 700 | GK1.5 | BD Biosciences |
| CD4 | APC-H7 | GK1.5 | BD Biosciences |
| CD8 | APC-H7 | 53-6.7 | BD Biosciences |
| CD44 | FITC | IM7 | BD Biosciences |
| CD62L | PE-Cy7 | MEL-14 | BD Biosciences |
| CD122 | PE | TM-b1 | BD Biosciences |
| CD127 | BV421 | A7R34 | BioLegend |
| KLRG1 | PE-eFluor 610 | 2F1 | Thermo Fisher Scientific |
| IFNγ | PE-CF594 | XMG1.2 | BD Biosciences |
| TNFα | PE-Cy7 | MP6-XT22 | BD Biosciences |
| IL-2 | VB421 | JES6-5H4 | BD Biosciences |
| PD-1 (CD279) | APC | J43 | BD Biosciences |
| CTLA-4 (CD152) | PE | UC10-4F10-11 | BD Biosciences |
| 2B4 (CD244) | FITC | 2B4 | BD Biosciences |
| CD160 | PE-CF594 | CNX46-3 | BD Biosciences |
| LAP | BV421 | TW7-16B4 | BioLegend |
| Foxp3 | FITC | FJK-16s | Thermo Fisher Scientific |
| CD25 | PE-Cy7 | PC61.5 | Thermo Fisher Scientific |
| IL-10 | Alexa Fluor 700 | JES5-16E3 | Thermo Fisher Scientific |
| RORγt | APC | AFKJS-9 | Thermo Fisher Scientific |
| IL-17A | PE-eFluor 610 | eBio17B7 | Thermo Fisher Scientific |
| IL-21 | PE | FFA21 | Thermo Fisher Scientific |

**Supplemental Table 2. Average of cell counts used for the flow cytometry analysis.**

|  | Group | CD3^+^ cells^ƒ^ | CD4^+^CD3^+^ cells^ƒ^ | CD8^+^CD3^+^ cells^ƒ^ |
| --- | --- | --- | --- | --- |
| 10 dpi | Y | 144065 | 54336 | 78588 |
|  | DA | 130522 | 84687 | 39759 |
| 30 dpi | Y | 100874 | 53935 | 40902 |
|  | DA | 85091 | 54917 | 26268 |
| 100 dpi | Y | 70655 | 32971 | 14982 |
|  | DA | 52057 | 34055 | 15248 |
| 260 dpi | Y | 118735 | 58009 | 38270 |
|  | DA | 111083 | 70763 | 34149 |
|  | Y/Y | 127656 | 75138 | 43713 |
|  | DA/Y | 121753 | 71187 | 44325 |

^ƒ^Averages of CD3^+^, CD4^+^CD3^+^, and CD8^+^CD3^+^ T cell count detected in each group of *T. cruzi* infected mice on days 10, 30, 100, and 260 days.
